# Targeted therapy for multiple myeloma: an overview on CD138-based strategies

**DOI:** 10.3389/fonc.2024.1370854

**Published:** 2024-04-09

**Authors:** Federico Riccardi, Carmela Tangredi, Michele Dal Bo, Giuseppe Toffoli

**Affiliations:** ^1^ Experimental and Clinical Pharmacology Unit, Centro di Riferimento Oncologico (CRO), Istituto di Ricovero e Cura a Carattere Scientifico (IRCCS), Aviano, Italy; ^2^ Department of Life Sciences, University of Trieste, Trieste, Italy

**Keywords:** multiple myeloma, CD138, shedding, immunotherapy, anti-cancer peptides

## Abstract

Multiple myeloma (MM) is an incurable hematological disease characterized by the uncontrolled growth of plasma cells primarily in the bone marrow. Although its treatment consists of the administration of combined therapy regimens mainly based on immunomodulators and proteosome inhibitors, MM remains incurable, and most patients suffer from relapsed/refractory disease with poor prognosis and survival. The robust results achieved by immunotherapy targeting MM-associated antigens CD38 and CD319 (also known as SLAMF7) have drawn attention to the development of new immune-based strategies and different innovative compounds in the treatment of MM, including new monoclonal antibodies, antibody-drug conjugates, recombinant proteins, synthetic peptides, and adaptive cellular therapies. In this context, Syndecan1 (CD138 or SDC1), a transmembrane heparan sulfate proteoglycan that is upregulated in malignant plasma cells, has gained increasing attention in the panorama of MM target antigens, since its key role in MM tumorigenesis, progression and aggressiveness has been largely reported. Here, our aim is to provide an overview of the most important aspects of MM disease and to investigate the molecular functions of CD138 in physiologic and malignant cell states. In addition, we will shed light on the CD138-based therapeutic approaches currently being tested in preclinical and/or clinical phases in MM and discuss their properties, mechanisms of action and clinical applications.

## 1 Introduction

Multiple myeloma (MM) is a hematological malignancy affecting plasma cells (PCs) lineage primarily found in the bone marrow (BM). The PCs are derived from B lymphocytes, a type of white blood cell in the immune system of most vertebrates that produces large amounts of antigen-specific immunoglobulins (Igs) in response to various stimuli (1). In most of patients affected by MM, PCs are transformed into cancerous cells, multiply in the BM, and secrete a large amount of a specific Ig (also known as monoclonal protein or M paraprotein), which accumulates in the blood and tissues and leads to organ damage and dysfunction (1, 2). To date, MM accounts for 1% of all cancers and about 10% of all hematological malignancies, making it the second most common hematological cancer after non-Hodgkin’s lymphoma (3). Several data show that the incidence of MM is strongly related to age and gender (1, 3, 4). In Italy, 38% of diagnoses affect people aged 70 and over and only 2% affect people younger than 40, with men being diagnosed with MM more frequently than women (www.airc.it). This cancer shows few characteristic signs that are driven by the production of M paraprotein and dysregulation in the network of signaling molecules produced by malignant PCs and other cellular components located in the BM microenvironment (BMME) (5). The most typical clinical manifestations that occur in MM patients are defined by the C.R.A.B. criteria, an acronym that stands for calcium elevation (or hypercalcemia), renal failure, anemia and/or bone disease with lytic lesions. This criteria defines specific signs and symptoms of end-organ damage that can be used to determine the stage of progression of MM and as an important tool for MM diagnosis and therapy (5, 6). Although standard chemotherapy regimens based on immunomodulatory drugs (IMiDs), proteasome inhibitors (PIs) or a combination of both have achieved remarkable results in prolonging the survival of MM patients, they also target a broad spectrum of healthy cells and therefore cause serious adverse events when administered (7, 8). To achieve a safer and durable response in MM patients several studies have identified key antigens overexpressed on malignant PCs essential for MM progression, and innovative targeted therapeutic strategies have been developed against them, mainly based on monoclonal antibodies (mAbs), immunoconjugates, synthetic peptides, and adaptive cellular therapies (see below). In addition to the widely studied MM-related antigens CD38 (9–11) and signaling lymphocyte activation molecule F7 (SLAMF7) (12–14), against which three mAbs have already received FDA approval for clinical use (10, 14), and the B-cell maturation antigen (BCMA) (15, 16), there is growing evidence for the key role of Syndecan1 (CD138 or SDC1), a transmembrane heparan sulfate proteoglycan (HPSG), in MM tumorigenesis (17–19). CD138 is highly expressed on malignant PCs and is involved in several cellular pathways responsible for their growth, survival, and proliferation by binding many growth factors and proteins in the extracellular matrix (ECM) (17, 20). In addition to the well-known cancer-related function of CD138 as an antigen receptor, its tumorigenic role is enhanced by its dysregulated shedding mechanism, which stimulates migration and angiogenesis of MM cells and overall correlates with poor prognosis and limited therapeutic efficacy (17–19). In this sense, the prominent role of CD138 in MM biology suggests the identification and development of novel therapeutic agents to suppress its activity, as no CD138-targeted therapy has yet been approved for clinical use in MM and only few are being investigated in relevant clinical phases. In this review, we aim to provide a brief overview of the most important molecular aspects of MM disease. Moreover, we focus our interest on CD138 and discuss its the role in MM tumorigenesis and progression. In addition, we provide a review of current CD138-based approaches in various phases of preclinical and clinical development, highlighting key information on their mechanisms of action, efficacy and safety profile.

## 2 Key molecular aspects of MM and the BMME associated to cancer

### 2.1 Ontogenesis of B cells

**Figure 1:**
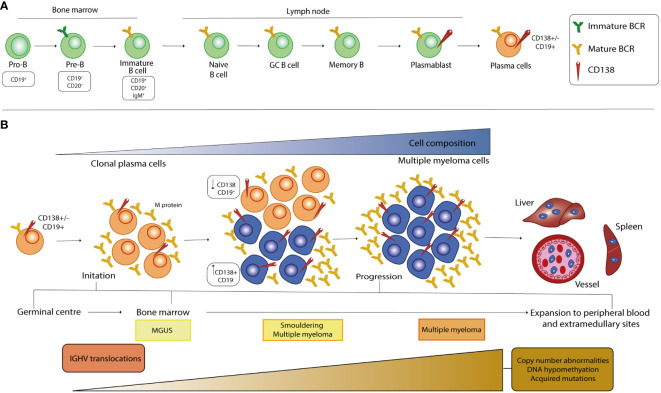
Stages of B cell development and pathogenesis of MM. **(A)** The development of B cells begins in the bone marrow, where the cells undergo a rearrangement of the heavy and light chains of immunoglobulins and express a mature BCR in the immature B cell stage. After the transition to the peripheral tissue, the B cells complete their maturation and during the final differentiation step, at the plasmablast stage, they acquire CD138 expression on their surface. **(B)** MM is a multistep process that starts from a premalignant stage called monoclonal gammopathy of undetermined significance (MGUS), in which long-lived plasmacells (LLPCs) begin to produce low levels of monoclonal protein (M-protein). When the amount of PC and the M-protein concentration increase, there is a transition from MGUS to an intermediate stage called smoldering multiple myeloma (SMM). At this stage, the malignant clone begins to increase CD138 cell surface expression, which inversely correlates with CD19 expression. The selection and spread of the malignant clone characterize the last stage of MM and causes infiltration of all organs, which correlates with poor prognosis. Primary genetic events in the development of MGUS, SMM and MM mainly include chromosomal translocations, whereas the number of secondary genetic alterations increases from MGUS to SMM and then to MM.

B lymphocytes originate from the hematopoietic stem cells (HSCs) in the BM and go through various stages of maturation to become a mature B cell after differentiation (**Figure 1A**). The development of B cells takes place primarily in the BM and continues in secondary lymphoid organs (SLOs) such as lymph nodes and spleen, where B cells can be activated into PCs. In the maturation in the BM, B cells pass through various developmental stages, each characterized by precise genetic patterns (21, 22). During this differentiation, B cells undergo V(D)J recombination and exhibit the pre-B cell receptor (pre-BCR) and eventually a mature BCR, which consists of the IgH-IgL complex and can bind antigens. B cells harboring the BCR on the cell membrane undergo positive or negative selection in the BM to prevent the further development of self-reactive cells and to ensure the formation of functional B cells. Clones that have passed both positive and negative selections, defined as transitional B cells, migrate to the spleen to complete their development and eventually mature into mature follicular B cells (FO) or marginal zone B cells (MZ), depending on the stimuli received by the BCR and other receptors (23, 24). After their differentiation, they are now referred to as mature B cells or naive B cells (25). Finally, the B cells migrate to SLOs where they undergo T cell-independent (MZ B cells) or -dependent (FO B cells) activation. When naïve B cells encounter their cognate antigen, they are activated and differentiate into short- and/or long-lived antibody-secreting PCs (SLPCs/LLPCs) after clonal expansion (26–28). SLPCs are proliferating cells with a lifespan of 3 to 5 days that are mainly formed in extrafollicular areas of SLOs and express IgM antibodies with low affinity for immediate protection. On the contrary, LLPCs are non-proliferating cells with a lifespan of several months to lifetime that are typically formed during the GC reaction and secrete high-affinity switch class antibodies located in BM cells (28). Two consecutive phases, which occur predominantly in FO and in GC, are required to the generation of LLPCs and memory B cells from the naïve B cell in the primary response to antigen. In the first step, the BCR-antigen interaction induces naïve B cells to differentiate into SLPCs and GC B cells in the B cell follicles. In the second step, the B cells are induced by antigens to differentiate into LLPCs and memory B cells in the GCs. In this way, memory B cells differentiate into LLPCs upon a recall response to antigens or by re-entering the GC reaction (29, 30). Normal PCs predominantly express CD19, CD45, CD27 and CD81 while malignant ones, undergoing MM transformation, show a characteristic down regulation of the mentioned markers but an overexpression of other antigens such as CD56, CD117, CD28, CD33, CD200 (31), GPRC5D and FcRH5 (32). In addition, particular key proteins viable for the development of therapeutic strategies against MM, including CD38, SLAMF7, BCMA and CD138, are present on the surface of healthy and malignant PCs, though mostly up regulated in the latter population (1, 33).

### 2.2 The development of MM is a multi-stage process driven by genetic mutations, pathways’ dysregulations and involves the interplay between the BMME and MM

Although a complete information regarding the events causative the MM pathogenesis is still missing, it is well known that this cancer is a progressive disease characterized by different stages of development, from the precursor disease state known as monoclonal gammopathy of undetermined significance (MGUS) to the asymptomatic smoldering MM (SMM) and MM. The string of events leading to the pathogenesis of MM begins with molecular alterations of mature B cells in the GC, a specialized microenvironment in the lymphoid follicle, and includes Ig translocations and hyperdiploidy, the most common form of aneuploidy in MM cells (34–37) (**Figure 1B**). Chromosomal translocations are mediated by errors in V(D)J recombination and place oncogenes (OGs) under the control of strong enhancers, such as the heavy-chain locus (IGH) on chromosome 14q32, and lead mainly to the dysregulation of D-cyclins and thus to the transition from G1 to S in the cell cycle (38). Premalignant PCs affected by these events are subjected to increased genomic instability, which leads to the acquisition of new alterations as MM progresses, mainly in the driver genes RAS, RB1 and MYC, and in the tumor microenvironment (TME) (35–38). Furthermore, the signaling pathways that are over-activated in MM have been largely described and support MM pathogenesis by promoting cell survival, proliferation, differentiation, and migration as well as angiogenesis, immune dysregulation, drug resistance mechanism and inhibition of apoptosis (39–53). The reciprocal interaction between MM cells and the BMME is crucial for the development and progression, especially in the early stages of malignant transformation of PCs, as well as for the treatment of the disease. The BM is composed of highly specialized cell lineages organized in anatomical and functional niches and harbors different cell types belonging either to the hematopoietic lineage, such as osteoclasts (OCs), B, T and natural killer (NK) lymphocytes and myeloid-derived suppressor cells (MDSCs), or to the non-hematopoietic cells, such as osteoblasts (OBs), stromal BM and endothelial cells (54–56). During the main course of MM evolution, malignant PCs show a tendency to homing to the BM compartments as they are dependent on the BMME signals for growth and survival (55, 57). In particular, the homing process involves the interaction of the CXC chemokine receptor type 4 (CXCR4) with its ligand, the chemokine CXCL12, which is thought to be crucial for the homing of plasmablasts in the BM and the survival of mature LLPCs in niches organized by CXCL12-expressing stromal cells (27, 55, 57). In MM, CXCR4 and CXCL12 proteins are upregulated on both PCs and stromal cells. The CXCL12-CXCR4 axis leads to an increase in the activity of very late antigen-4 (VLA-4 or α4β1), an integrin located on the MM cell membrane that enhances its binding to fibronectin and its ligand vascular cell adhesion molecule 1 (VCAM-1), the latter being expressed on the surface of the endothelial cells of the BM microvasculature. P-selectin and E-selectin contribute to the anchoring process in the initial stages of the process (58–62). The interaction between accessory components of BM and malignant cells leads to dysregulated activation of key signaling cascades by affecting the production of cytokines and chemokines in stromal, bone and immune cells, initiating or altering physical interactions via adhesion molecules, and inducing the biosynthesis of exosomes, small membrane-based vesicles that serve as a means of transport for bioactive molecules (39, 54).

### 2.3 Epigenetic abnormalities contribute to MM oncogenesis, progression and drug resistance

As in many pathological conditions, epigenetic alterations involving DNA methylation, histone modifications and non-coding RNAs (ncRNAs) regulation play an important role in the pathogenesis of MM (63). Abnormal deposition of epigenetic markers and up and downregulation of ncRNAs likely dysregulate the expression pattern of key tumor suppressor genes (TSGs) and OGs involved in cell cycle control, apoptosis and cellular differentiation, thus contributing to disease progression (63–66). Furthermore, by Next Generation Sequencing (NGS) analyses non-synonymous point mutations of epigenetic regulators such as lysine demethylases KDM6A/UTX and KDM6B/JMJD3, histone methyltransferases MMSET and MLL and homeobox protein Hox-A9 (HOXA9) have been uncovered in MM cells (67, 68). The mechanisms responsible for dysregulated DNA methylation in MM cells are not yet fully characterized but are mainly caused by aberrant expression of DNA methyltransferases. Decreased expression of these enzymes leads to DNA hypomethylation, which is increased in MM cells and associated with the expression of several cancer-related genes involved in cell migration (69), proliferation (70) and drug resistance mechanisms (71), ultimately leading to myelomagenesis and poor survival. In addition, DNA hypermethylation was observed at the CpG islands in the promoter of specific TSGs, including some that are critical for cell cycle progression (72–74) and cell adhesion (75, 76). Hypermethylation of genes’ promoter has been also associated with activation of the WNT signaling pathway (69, 77), an aggressive phenotype, poorer response to therapies and short survival for patients (78). Furthermore, epigenetic changes in MM can be influenced by the TME and are crucial in explaining the high degree of plasticity and clonal heterogeneity in different tumors (79, 80). Histones undergo acetylation and methylation, two well-known post-translational modifications (PTMs) that are largely involved in the modulation of gene expression. In MM, deregulation of histone methylation modifiers is associated with aberrant chromatin accessibility and consequently with abnormal cell growth, proliferation, adhesion and therapeutic potential (81–83). Similarly, histone acetylation modifiers have been found to have carcinogenic significance as they are involved in cell proliferation, survival and apoptosis (84–86). Recently, a differential analysis of histone mark profiles highlighted links between histone modifications, including H3K9me3, H3K27me3 and H3K4me3, and cytogenetic abnormalities or recurrent mutations in MM cells, suggesting their prognostic value and association with drug response (87). Since DNA methylation and histone modifications have been reported to modulate the levels of microRNAs (miRNAs) (88), epigenetic changes could also be responsible for impaired miRNAs expression. In the panorama of ncRNAs, miRNAs are probably the most important elements in the regulation of gene expression at the post-transcriptional level. Several miRNAs are present in cells, and a consistent fraction is deregulated in malignant cells, supporting oncogenesis and progression of MM (89). Since miRNAs exert a physiological negative regulation of gene expression, MM exploits this mechanism to thrive by reducing the expression of certain miRNAs targeting cancer-relevant OGs to enhance their level, while increasing the amount of specific miRNAs to inhibit the expression of TSGs, thus blocking their anti-cancer effects (90, 91). As with other markers, the altered expression of key miRNAs in malignant PCs can be used as a prognostic and diagnostic tool, as in the case of miR-203 (92) or miR-1246 (93), or to better understand the underlying mechanisms leading to transformation of PCs and drug response and resistance in MM.

### 2.4 Dysregulation of the immune system within the BMME in MM

Since the BM is an immunological tissue, it harbors many immature and mature cellular components of the innate and adaptive immune system. These immune cells originate either from the myeloid compartment, such as MDSCs, macrophages and dendritic cells (DCs), or from the lymphoid compartment, which gathers helper (CD4+) and cytotoxic (CD8+) T lymphocytes, NK and B cells. Progression of the disease to more advanced stages is accompanied by severe immunologic dysfunction in the BMME of MM patients and requires the cooperation of members of both compartments (54, 94–98). In general, immune tolerance to immune surveillance is promoted by different mechanisms, including reprogramming of macrophages, generation of T cell memory, activation of dendritic and T cells and alteration of immune checkpoint molecules through the production of various soluble factors (54, 94–98). Although it has been repeatedly demonstrated that MDSCs modulate the CD8+ T response by activating regulatory T cells while inhibiting effector T cells, and that macrophages respond to pro-tumor chemokines directing their polarization to M2 macrophages, including subsets of tumor-associated macrophages (TAMs), most dysregulation occurs in the lymphoid compartment (54, 94–98). For instance, CD4+ T cells go toward distribution and functional abnormalities and trigger antitumor responses by interacting with macrophages in the BM, while CD8+ T cells increase in MM but show low proliferation and cytotoxic activity against MM cells and approach exhaustion. NK and B cell activities are severely impaired in MM due to reprogramming of activating/inhibitory receptors for NK and impaired functionality for B cells. Finally, mesenchymal stromal cells (MSCs) contribute to the creation of an immunosuppressive environment (54, 94–98).

## 3 Syndecan1 is markedly involved in MM pathogenesis

### 3.1 The Syndecans protein family mediates cell signaling and biological functions

**Figure 2:**
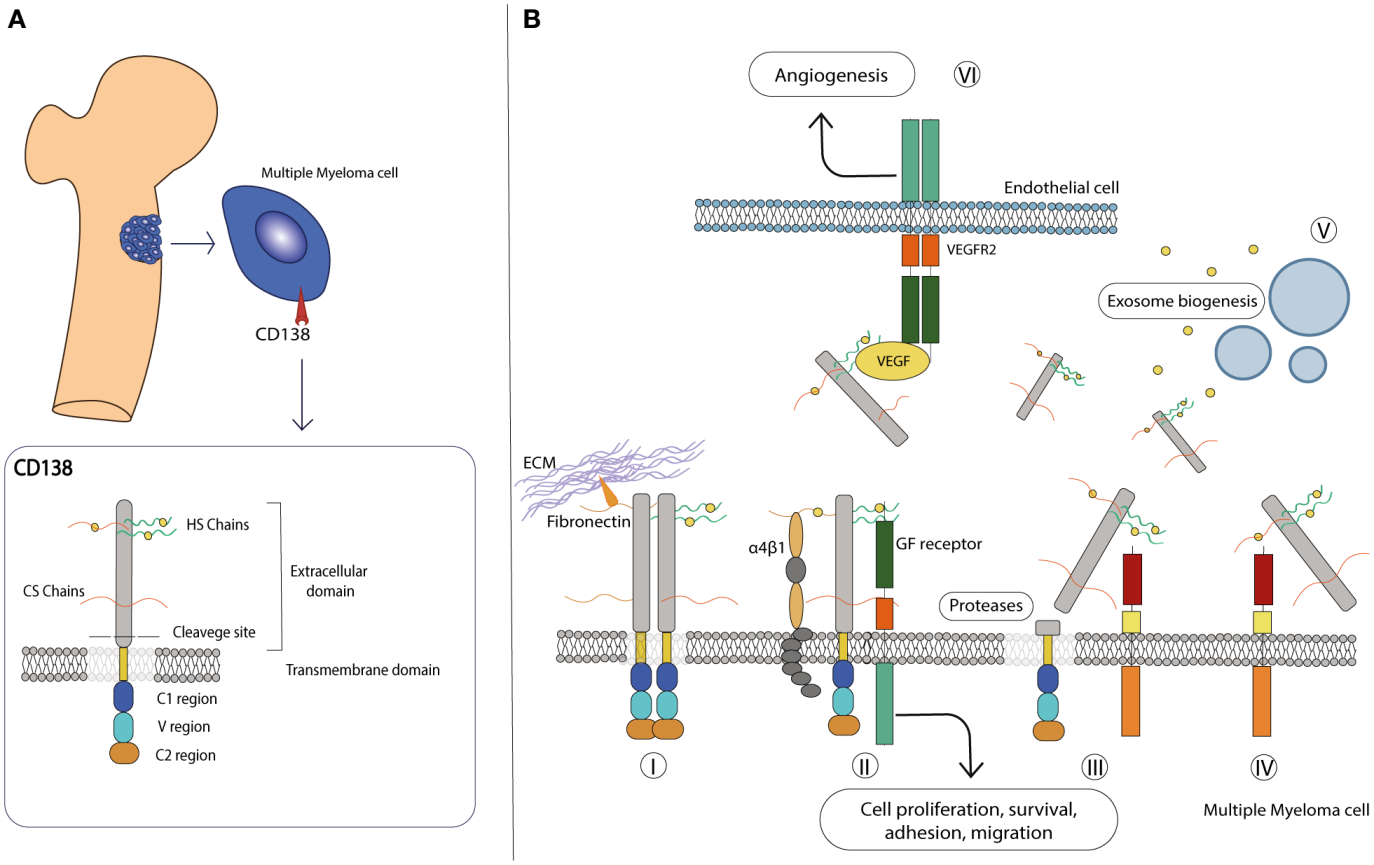
CD138 molecular structure and role in the microenvironment. **(A)** Schematic representation of the molecular structure of CD138. Details on each domain are provided in the text. **(B)** CD138 has various roles in the MM microenvironment: (I) it interacts with various extracellular matrix, proteins such as laminin and fibronectin, and promotes adhesion and migration. (II) It acts as a co-receptor in various interactions, stabilizes multicomplex formation between GF receptor and α4β1 integrin, and activates pathways involved in cell proliferation and survival. (III) Its extracellular domain can be cleaved by proteases and released in the extracellular environment, (IV) also acting as a co-receptor on other cells and activating paracrine signaling. (V) With the transmembrane domain, CD138 activates various factors, including those involved in exosome biogenesis. (VI) On endothelial cells, it facilitates the interaction between VEGF and VEGFR2, leading to activation of the angiogenic signaling pathway.

The ECM consists of an intricate, non-cellular network of carbohydrates and proteins that surrounds cells and whose main function is to provide them with the necessary biochemical and structural support. Although the composition of the ECM varies according to multicellular structures, it is generally composed of several fibrous proteins, proteoglycans (PGs) and other molecules arranged in a reticular mesh-like structure. The contribution of the ECM to important cellular processes required for the maintenance of tissue homeostasis, including cell signaling, proliferation, differentiation and migration, has been extensively studied (99–101). Syndecans (SDCs) are a family of type I transmembrane HSPGs composed of sulfated glycosaminoglycans (GAGs), heparan sulfate (HS), or both HS and chondroitin sulfate (CS) covalently linked to core proteins (19, 102). The HS polysaccharide chains confer an important modulatory role to SDCs in various processes by interacting with the “heparin-binding domains” harbored by a variety of growth factors (GFs), including Epidermal growth factor (EGF), Fibroblast growth factor (FGF), Hepatocyte growth factor (HGF), Platelet growth factor (PDGF) and Vascular endothelial growth factor (VEGF), but also present on proteases and various other ligands in the ECM compartment (103–105). This engagement leads to the triggering of downstream signal transduction pathways that ultimately alter cell behavior in response to specific stimuli (106). In mammals, the SDC family is composed of four members, from SDC1 to SDC4, which share common features in terms of composition and function. All members consist of three different structural domains: the extracellular, the transmembrane and the cytoplasmic domains (18, 102) (**Figure 2A**). The first domain harbors the HS and CS chains, which are covalently bound to serine residues, and mediates most interactions with a variety of ligands. In addition, the extracellular domain contains the cleavage site, a specific SDC portion located in the proximity of outer membrane that is recognized by specific proteases and allows the release of a functional soluble ectodomain (see below). The single transmembrane domain anchors the SDCs to the membrane and carries the consensus G-X-X-X-G, a conserved dimerization motif. Finally, the cytoplasmic tail is divided into two conserved regions (C1 and C2) flanked by a variable region (V). The C1 region is located under the cell membrane, where it interacts with the cytoskeleton and is involved in cytoskeletal organization and endocytosis, while the C2 region, the C-terminal domain, can bind various adaptor proteins that harbor the PDZ domains and mediate growth factor-induced cascades. Interestingly, C1 and C2 are highly conserved among SDCs, while the V region shows little similarity, although it is highly conserved between species (17, 18).

### 3.2 The role of CD138 in physiological state

Among the members of the SDC family, SDC1 (also known as CD138) is the most studied gene since it was biochemically characterized in the murine mammary epithelial NMuMg cell line by Merton Bernfield and colleagues in 1989 (107). The human CD138 gene is located on chromosome 2 (2p24.1) and has a length of approximately 24.6 kb, divided into 5 exons, and encodes the HSPG protein CD138. It has a predicted molecular size of 33 kDa and consists of 283 amino acids (107), although its mass is much larger due to its carbohydrate modifications - three sites for CS chains and two for HS chains - in the extracellular domain (108). Together with SDC2 and SDC4, it is translated with a signal peptide that is later cleaved during its processing (102). CD138 is mainly expressed on epithelial cells, transiently on developing mesenchymal cells and mature B cells and together with other molecules maintains the normal morphological phenotype (18, 109). Thanks to the presence of HS outer chains that facilitate ligand-receptor recognition and its ability to interact with integrins and ECM components such as fibronectin and laminin, CD138 serves as a key receptor for the maintenance of physiological cell state (110) (**Figure 2B**). Like other SDC members, CD138 undergoes shedding, a regulated proteolytic cleavage in which a portion of the extracellular domain is cleaved off at a juxtamembrane site and released as soluble form (111, 112). Shedding is usually mediated by sheddases, a general term that includes members of the matrix metalloproteinase (MMP) (113), A-disintegrin and metalloproteinase (ADAM) (114) and Beta-site amyloid precursor protein-cleaving enzyme 1 (BACE) protein families (115). In addition, heparanase (HPSE), an endoglycosidase that acts within the ECM to trim HS chains and generate specific short fragments by exposing the core protein for cleavage by MMPs, is largely involved in CD138 shedding and is severely implicated in tumor angiogenesis, growth and metastasis (116–118). After cleavage, the newly formed fragment is deposited in the ECM environment where it exerts autocrine and paracrine effects by reducing the available binding sites on the transmembrane of CD138 and other PGs, acting as a sponge for cytokine/chemokine/GFs ligands and consequently affecting downstream cascades (17, 111). In addition, the shed fragment can also form morphogen gradients across tissues and penetrate the cytoplasmic environment of the cells, indirectly modulating the gene expression patterns of selected genes (119). This process occurs to a small extent constitutively but can be significantly altered in response to various stimuli and/or under pathological conditions, making CD138 a diagnostic and prognostic marker for monitoring infectious states and tumor progression. Nevertheless, the involvement of CD138 in cell-matrix interactions, cell migration and proliferation makes it a valuable player in the progression of pathological conditions, including inflammation and cancer (17–19, 102).

### 3.3 The role of CD138 in MM pathological condition

CD138 is highly expressed on both end-stage differentiated normal and malignant PCs and plays a causal role in the progression of MM, as evidenced by the fact that its ablation leads to poor growth of MM cells *in vitro* and apoptosis *in vivo* (120, 121). In the context of malignant transformation, it has been reported that CD138 expression correlates with disease severity (33). The correlation between its low epithelial expression and a high shed level in serum, which is mainly favored by dysregulated HPSE activity, indicates a high tumor stage with a low overall survival rate (122). However, this correlation can also exist at the time of diagnosis, being a sign of a poor prognosis, and used as an index of the effectiveness of the treatment regimen (17, 33). Accordingly, patients who respond to chemotherapy have lower CD138 serum levels than patients who do not respond to treatment, emphasizing the key role of CD138 in MM progression (123). Moreover, CD138 surface expression has been shown to dynamically drive the switch between growth and spread of MM depending on nutrient conditions (124), to also act as a co-receptor for transmembrane activator and CAML interactor (TACI) and APRIL, promoting the APRIL/TACI-associated pathways that induce proliferation and survival of cancerous PCs (125), and few studies have addressed the role of CD138 in bone disease, one of the pathognomic hallmarks of MM pathogenesis (50, 126, 127). The core of MM-related bone disease lies in the presence of an unbalanced equilibrium in physiologic bone turnover, with an increase in bone resorption activity by OCs and a concomitant decrease in bone formation mediated by OBs. This phenomenon has been shown to be mediated by MM cells which, by interacting with bone cells and inducing the production of cytokines from the BMME, stimulate the OCs activity and in parallel suppresses OBs function, promoting disease progression (128, 129). The increased OCs activity is primarily mediated by the RANK/RANKL/OPG molecular pathway, which is critical for their maturation and bone remodeling in both physiological and pathological conditions. The key components of this pathway are RANK, a transmembrane receptor belonging to the tumor necrosis factor receptor (TNFR) molecular subfamily expressed on precursor and mature OCs, and its ligand RANKL, a cytokine expressed by OBs (51, 127, 130). The interaction between RANK/RANKL is of fundamental importance for the differentiation, activation and survival of OCs and significantly controls bone resorption. However, OPG, a cytokine secreted by OBs and BMSCs, disrupts RANK/RANKL binding by acting as a soluble decoy for RANKL and plays a critical role in inhibiting osteoclastogenesis and consequently excessive bone resorption by OCs (130, 131). In MM, malignant PCs act on cellular cascades to stimulate the expression of RANKL and decrease the availability of OPG within the BMME, shifting the RANKL/OPG balance in favor of OPG. In this way, the PCs cause an increase in the number and activity of OCs to the detriment of OBs, leading to bone destruction and disease. Furthermore, upregulation of CD138 by MM cells enables binding, internalization and degradation of OPG, thereby promoting RANKL-mediated activity of OCs and osteolysis (127, 130, 131). Similarly, Activin A is a pleiotropic cytokine that belongs to the TGF-β superfamily and can trigger the NF-kB signaling pathway by inducing RANK expression to support OCs differentiation (132). Overall, RANKL and its antagonist OPG are two important molecules to understand the features of myeloma bone disease (MBD) as they are closely associated with the clinical outcome of the disease (133). Finally, CD138 in synergy with HPSE has been shown to promote MM bone disease by activating the HGF-Met-IL-11-RANKL signaling axis, resulting in inhibition of bone formation and promotion of bone resorption (134). Of note, a small subpopulation of human MM cells lacking the expression of CD138 has been reported to have tremendous proliferative potential, drug resistance, carcinogenic ability, and the capacity to differentiate into CD138+ PCs in *in vitro* experiments and *in vivo* models (135, 136). In line with these findings, patients with CD138- plasmacytomas have been diagnosed with worse prognosis (124). Additionally, Wu and colleagues found a dramatic difference in gene expression between CD138- and CD138+ PCs, mainly centered on the ataxia telangiectasia mutated and Rad3 related kinase-checkpoint kinase 1 (ATR-CHK1) cell cycle pathway, which is closely related to the clonal proliferation characteristics of CD138+ PCs and correlates with low overall survival in MM patients (120). Nevertheless, there are few studies addressing the role of epigenetic regulators in CD138+ PCs. Zhang and colleagues have found DNA methylation peaks in intragenic and intronic regions in bone marrow-derived CD138+ MM cells and found hypermethylation-mediated inhibition of tumor suppressors miRNA-10b-5p and miRNA-152, leading to overexpression of their target genes OGs DNMT1, BTRC, MYCBP, and E2F3 (137). In addition, Gullà and colleagues showed that upregulation of protein arginine methyltransferase 5 (PRMT5), an enzyme involved in growth and survival pathways that promote tumorigenesis, was closely associated with decreased progression-free survival (PFS) and overall survival (OS) in immunopurified CD138+ cells, suggesting the oncogenic role and prognostic significance of PRMT5 in MM pathogenesis (138). Notably, Yan and colleagues demonstrated that piRNA-823, a member of piwi-interacting RNAs, a large class of endogenous small ncRNAs potentially involved in post-transcriptional gene silencing, contributes to tumorigenesis by regulating *de novo* DNA methylation and angiogenesis in primary CD138+ MM cells (139).

### 3.4 CD138 shedding is a regulated mechanisms in MM and associated to tumorigenesis

The importance of CD138 shedding in various cancers/hematological malignancies is well documented (19, 33, 140), including in MM cells and within dysfunctional BMME, and unfortunately associated with poor prognosis, treatment and overall survival once its serum level increases in advanced-stage disease (116, 141–145). In CD138 shedding, HPSE plays a key role as its dysregulated expression correlates with poor prognosis in MM patients (116, 146). Several studies have addressed the relationship between CD138 shedding, HPSE and the progression of MM (117, 142, 146, 147). For example, upregulation of HPSE promotes CD138 shedding and expression, which is responsible for the increased concentration of CD138 ectodomain in BMME and MM serum, thus promoting angiogenesis, growth, and metastasis of MM to bone *in vivo* (117). Similarly, Purushothaman and colleagues observed that in MM, the HPSE-induced increase in CD138 shedding is facilitated by the sustained stimulation of ERK phosphorylation, which in turn drives MMP-9 expression (147). In addition, Ramani and co-workers reported that HPSE stimulates an increase in the expression of HGF, a putative paracrine and autocrine regulator of MM growth, and CD138 shedding to enhance HGF signaling in the MM environment, fostering its critical role as a modulator of MM progression (146). Similarly, it was observed that soluble CD138 binds to HGF via HS chains remaining on the fragment and that this interaction promotes activation of the PI3K/Akt and Ras/Raf/MAPK/ERK kinase signaling pathways as well as stimulation of the HGF/Met pathway, leading to proliferation and survival of MM cells (142). The involvement of CD138 and/or HPSE in other important signaling cascades has been investigated as well (148–150). In particular, CD138 promotes the angiogenic phenotype of MM endothelial cells by supporting VEGF-VEGF2 signaling (148) and the binding of EGF family ligands to the HS chains of CD138 is essential for the growth of MM (150). In addition, Purushothaman and colleagues also demonstrated that an increase in HPSE expression by enhancing VEGF and other factors leads to a decrease in nuclear CD138 and increased histone acetyltransferase (HAT) activity, which in turn upregulates the transcription of several genes that cause an aggressive tumor phenotype in MM (149).

## 4 CD138-based therapeutic strategies in MM

### 4.1 The development of CD138-based therapies is associated with benefits and risks

**Figure 3:**
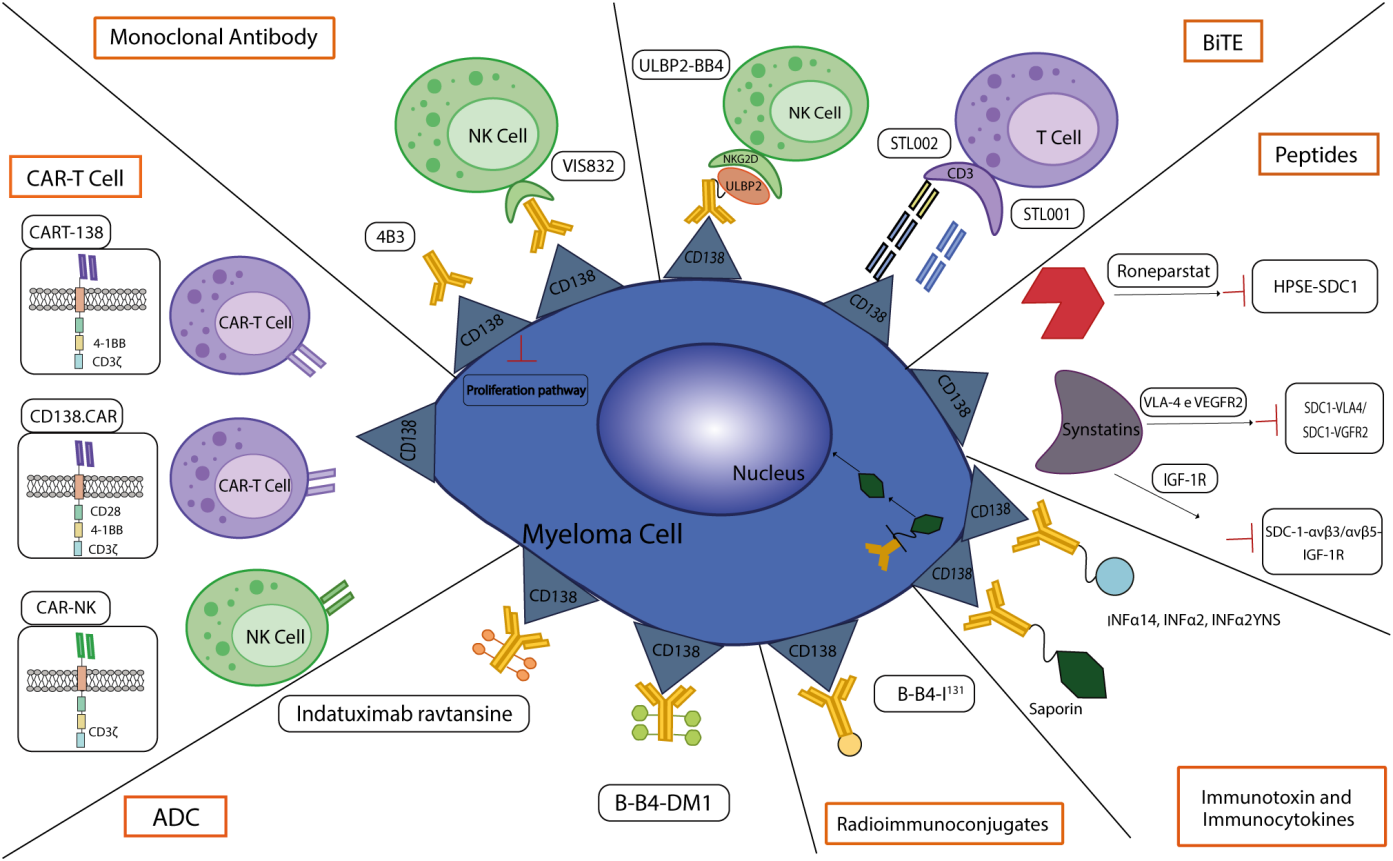
CD138-based therapies. Schematic representation of CD138-based therapies against MM.

In the next future, CD138-based therapies could gain a firm place in the panorama of MM therapeutic strategies. Many studies are investigating the use of CD138 as a suitable target for therapy as it is readily accessible on the cell membrane and it is fundamental in the physiology of malignant PCs, especially in aggressive relapsed/refractory (RR) stages of MM (**Table 1**). Similarly to other immunotherapeutic approaches, CD138-based strategies are associated with some benefits but also with some disadvantages and risks. Most of the current anti-CD138 therapeutics are developed by engineering and/or arming mAbs with toxic payloads, modifying T/NK cells to recognize CD138+ clones and synthesizing short peptides (**Figure 3**), and some of them provided remarkable results in terms of PCs targeting, therapeutic efficacy and safety (see below). The exceptional accuracy in the targeting of CD138 on MM cells can significantly reduce the systemic toxicities associated with conventional chemotherapy and stimulate the patient’s immune system to efficiently recognize and attack malignant cells, potentially leading to long-term positive benefits. The eradication of CD138+ clones can be achieved by direct/indirect mechanisms elicited by mAbs (VIS832), ADCs (indatuximab ravtansine) and other immunoconjugates (B-B4-I^131^), or by activating CD138-specific T cells to elicit strong cytotoxic effects against malignant cells, as is the case of PVX-410 cancer vaccine and CART-138 cells (see below). In addition, some studies have shown the possibility of combining immunotherapy with other anti-MM agents, such as the chemotherapeutic agents widely used in the treatment of MM, leading to encouraging results in terms of PFS and OS in patients (151, 152). Nonetheless, the development of CD138-based strategies must deal with complexities and uncertainties. Like other antigen-based approaches, CD138-based therapy may not be effective in all patients. Response may depend on environmental and individual factors, such as the health of the immune system, which may be fatally compromised in already debilitated MM patients, and the specific molecular characteristics of the malignant cells (158). For example, higher doses may be necessary to achieve a therapeutic response, but at the same time may cause immune-related side effects and severe or potentially fatal toxicities, leading to poor treatment outcome and, in the worst case, accelerating patient death. Besides, a fraction of patients may only have a partial response to therapy even after the administration of higher doses of the compound, with the potential manifestation of non-specific side effects (158). Moreover, a known common feature of cancer is its ability to overcome the efficacy of therapeutic approaches by deploying drug resistance mechanisms (159). Consequently, as therapy progresses, some fractions of MM clones may evade the benefits of treatment by either downregulating the expression of CD138 or gradually reducing its presence in the cell membrane, eventually leading to treatment failure and disease progression in some patients. In addition, some therapies may require a long time to be developed or be too expensive from an economic perspective and therefore inaccessible to most of the patients or healthcare systems (160). Furthermore, although most CD138-based strategies have provided encouraging data *in vitro* and *in vivo*, only few of them are under evaluation in relevant clinical phases (**Table 2**), meaning that long-term results on their efficacy and safety are still undisclosed and further research is needed to fully understand their potential benefits and limitations.

**Table 1:** Clinical trials in MM.

NCT Number	Title	Status	Conditions	Interventions	Phases	Refs
NCT03196414	Study of T Cells Targeting CD138/BCMA/CD19/More Antigens (CART-138/BCMA/19/More) for Chemotherapy Refractory and Relapsed Multiple Myeloma	Recruiting	MM	Biological: CART-138/BCMA/19/more	Phase 1|Phase 2	–
NCT03672318	Study of ATLCAR.CD138 Cells for Relapsed/Refractory Multiple Myeloma	Recruiting	MM|Immune System Diseases	Drug: CAR138 T Cells	Phase 1	–
NCT01745588	Autologous Stem Cell Transplant With Pomalidomide (CC-4047¬Æ) Maintenance Versus Continuous Clarithromycin/ Pomalidomide / Dexamethasone Salvage Therapy in Relapsed or Refractory Multiple Myeloma	Active, not recruiting	MM	Drug: Pomalidomide|Procedure: stem cell|Drug: Dexamethasone|Drug: Clarithromycin	Phase 2	–
NCT06006741	Universal CAR-T Cells Targeting Multiple Myeloma	Recruiting	MM in Remission	Biological: MM-specific universal CAR T cells	Phase 1	–
NCT00869232	UARK 2008-02 A Trial for High-risk Myeloma Evaluating Accelerating and Sustaining Complete Remission	Active, not recruiting	MM	Drug: Velcade|Drug: Melphalan|Drug: Thalidomide|Drug: Dexamethasone|Drug: Cisplatin|Drug: Adriamycin|Drug: Cyclophosphamide|Drug: Etoposide	Phase 2	–
NCT05759793	A Study of CAR-GPRC5D in Patients With Relapsed/Refractory Multiple Myeloma or Plasma Cell Leukemia	Recruiting	RRMM|PCL	Drug: CAR-T (CAR-GPRC5D)	Phase 1	–
NCT04850846	Investigation of Metformin for the Prevention of Progression of Precursor Multiple Myeloma	Recruiting	MGUS|SMM	Drug: Metformin XR|Other: Placebo	Phase 2	–
NCT04790474	Ixazomib-pomalidomide-dexamethasone as Second or Third-line Combination Treatment for Patients With Relapsed and Refractory Multiple Myeloma Previously Treated With Daratumumab, Lenalidomide and Bortezomib	Recruiting	RRMM	Drug: ixazomib-pomalidomide-dexamethasone	Phase 2	–
NCT04975997	Open-label Study Comparing Iberdomide, Daratumumab and Dexamethasone (IberDd) Versus Daratumumab, Bortezomib, and Dexamethasone (DVd) in Participants With Relapsed or Refractory Multiple Myeloma (RRMM)	Recruiting	MM	Drug: Dexamethasone|Drug: Daratumumab|Drug: Bortezomib|Drug: Iberdomide	Phase 3	–
NCT02773030	A Study to Determine Dose, Safety, Tolerability, Drug Levels, and Efficacy of CC-220 Monotherapy, and in Combination With Other Treatments in Participants With Multiple Myeloma	Active, not recruiting	MM	Drug: CC-220|Drug: Dexamethasone|Drug: Daratumumab|Drug: Bortezomib|Drug: Carfilzomib	Phase 1|Phase 2	–
NCT03651128	Efficacy and Safety Study of bb2121 Versus Standard Regimens in Subjects With Relapsed and Refractory Multiple Myeloma (RRMM)	Active, not recruiting	MM	Biological: bb2121|Drug: Daratumumab|Drug: Pomalidomide|Drug: Dexamethasone|Drug: Bortezomib|Drug: Ixazomib|Drug: Lenalidomide|Drug: Carfilzomib|Drug: Elotuzumab	Phase 3	–
NCT01946477	Pomalidomide in Combination With Low-dose Dexamethasone or Pomalidomide in Combination With Low-dose Dexamethasone and Daratumumab in Subjects With Relapsed or Refractory Multiple Myeloma Following Lenalidomide-based Therapy in the First or Second Line Setting	Active, not recruiting	MM	Drug: Pomalidomide|Drug: Dexamethasone|Drug: Daratumumab	Phase 2	–
NCT03896737	Daratumumab-bortezomib-dexamethasone (Dara-VCd) vs Bortezomib-Thalidomide-Dexamethasone (VTd), Then Maintenance With Ixazomib (IXA) or IXA-Dara	Active, not recruiting	MM	Drug: Daratumumab plus Velcade Cyclophosphamide Dexamethasone|Drug: Velcade Thalidomide Dexamethasone	Phase 2	–
NCT00723359	Safety and Dose Determining Study of BT062 in Patients With Relapsed or Refractory Multiple Myeloma	Completed	MM	Drug: BT062	Phase 1	(151–153)
NCT01001442	Safety and Dose Determining Multi-dose Study of BT062 in Patients With Relapsed or Refractory Multiple Myeloma	Completed	MM	Drug: BT062	Phase 1|Phase 2	
NCT01638936	BT062 in Combination With Lenalidomide or Pomalidomide and Dexamethasone in Patients With Multiple Myeloma	Completed	MM	Drug: BT062, intravenous administration	Phase 1|Phase 2	
NCT01296204	Radioimmunotherapy (RIT) in MULTIPLE MYELOMA Using the Antibody B-B4 Radiolabelled With IODE 131	Completed	MM	Drug: BB4 antibody-Iodine 131	Phase 1	(154)
NCT01886976	Treatment of Chemotherapy Refractory Multiple Myeloma by CART-138	Unknown status	MM	Biological: CART-138 cells	Phase 1|Phase 2	(155)
NCT01718899	Phase 1/2a Study of Cancer Vaccine to Treat Smoldering Multiple Myeloma	Completed	SMM	Biological: PVX-410	Phase 1	(156)
NCT02886065	A Study of PVX-410, a Cancer Vaccine, and Citarinostat +/- Lenalidomide for Smoldering MM	Active, not recruiting	SMM	Drug: Hiltonol|Drug: Citarinostat|Drug: Lenalidomide|Biological: PVX-410	Phase 1	–
NCT01764880	SST0001 (Roneparstat) in Advanced Multiple Myeloma	Completed	MM	Drug: SST0001 (Roneparstat)	Phase 1	(157)

Clinical trials present on ClinicalTrials.gov searching the keywords “multiple myeloma” and “138” in “all studies”. The research has been done adding the filters: recruiting, active, not recruiting, early phase 1, phase 1, phase 2 and phase 3. The research has been performed on January 5^th^, 2024. Trials going from “NCT00723359” to “NCT01764880” have been added later with the references found in the manuscript. MM: multiple myeloma. SMM, smoldering multiple myeloma; MGUS, monoclonal gammopathy of undetermined significance.

**Table 2:** CD138-based therapies in MM.

Strategy	Treatment	NCT Number	Study Title	Developmental state	Study Status	Phases	Refs
mAbs	VIS832	–	–	Preclinical	–	–	(161, 162)
	4B3	–	–	Preclinical	–	–	(163)
ADCs	Indatuximab ravtansine (BT062)	NCT00723359	Safety and Dose Determining Study of BT062 in Patients With Relapsed or Refractory Multiple Myeloma	Clinical	Completed	PHASE1	(151–153)
	Indatuximab ravtansine (BT062)	NCT01001442	Safety and Dose Determining Multi-dose Study of BT062 in Patients With Relapsed or Refractory Multiple Myeloma	Clinical	Completed	PHASE1/2a	(151–153)
	Indatuximab ravtansine (BT062)	NCT01638936	BT062 in Combination With Lenalidomide or Pomalidomide and Dexamethasone in Patients With Multiple Myeloma	Clinical	Completed	PHASE1/2a	(151–153)
	B-B4-DM1	–	–	Preclinical	–	–	(164)
Radioimmunoconjugates	B-B4-Iodine-131	NCT01296204	Radioimmunotherapy (RIT) in MULTIPLE MYELOMA Using the Antibody B-B4 Radiolabelled With IODE 131	Clinical	Completed	PHASE1	(154)
	B-B4-Bismuth-213	–	–	Preclinical	–	–	(165)
	B-B4-Bismuth-213-9E7.4	–	–	Preclinical	–	–	(166, 167)
	B-B4-Lutetium-177-9E7.4	–	–	Preclinical	–	–	(167)
Immunotoxins	B-B2-saporin	–	–	Preclinical	–	–	(168)
	B-B4-saporin	–	–	Preclinical	–	–	(168)
Immunocytokines	Anti-CD138-IFNα14	–	–	Preclinical	–	–	(169, 170)
	Anti-CD138-IFNα2	–	–	Preclinical	–	–	(169, 170)
	Anti-CD138-IFNα2YNS	–	–	Preclinical	–	–	(169, 170)
BsAbs	ULBP2-BB4	–	–	Preclinical	–	–	(171)
	STL-001	–	–	Preclinical	–	–	(172)
	STL-002	–	–	Preclinical	–	–	(173)
CAR-Ts	CART-138	NCT01886976	Treatment of Chemotherapy Refractory Multiple Myeloma by CART-138	Clinical	Unknown	PHASE1/2a	(155)
	CD138.CAR	NCT03672318	Study of ATLCAR.CD138 Cells for Relapsed/​Refractory Multiple Myeloma	Clinical	Recruiting	PHASE1	(174)
	CAR-NK	–	–	Preclinical		–	(175)
Cancer vaccines	PVX-410	NCT01718899	Phase 1/2a Study of Cancer Vaccine to Treat Smoldering Multiple Myeloma	Clinical	Completed	PHASE1/2a	(156)
	PVX-410	NCT02886065	A Study of PVX-410, a Cancer Vaccine, and Citarinostat +/- Lenalidomide for Smoldering MM	Clinical	Active, not recruiting	PHASE1	–
SSTNs	SSTN-IGF-1R	–	–	Preclinical	–	–	(176, 177)
	SSTN-VLA4	–	–	Preclinical	–	–	(176, 177)
	SSTN-VEGFR2	–	–	Preclinical	–	–	(176, 177)
HPSE inhibitor	Roneparstat (SST0001)	NCT01764880	SST0001 (Roneparstat) in Advanced Multiple Myeloma	Clinical	Completed	PHASE1	(157)

The research has been performed on March 18^th^, 2024.

### 4.2 Monoclonal antibodies

Immunotherapy with mAbs is widely used to treat a variety of solid and hematological cancers, not only as monotherapy but also in combination with conventional chemotherapeutic agents and is clinically effective also on patients with advanced-stage cancers. mAbs exert potent anti-tumor activity by interacting with components of the immune system to induce effector functions through antibody-dependent cell-mediated cytotoxicity (ADCC), complement-dependent cytotoxicity (CDC) and antibody-dependent cellular phagocytosis (ADCP) and/or affect tumor biology through direct modulation of survival-related signal transduction (178).

#### 4.2.1 VIS832

VIS832 is an afucosylated humanized IgGk mAb derived from the B-B4 clone. This mAb showed robust and dose-dependent CD138 engagement to MM cell lines and patient-derived MM cells, as well as target-specific ADCC mediated by NK cells and macrophages against MM cell lines and autologous patient cells. Further *in vitro* experiments showed that VIS832 enhanced lysis of MM cells in combination with the IMiDs lenalidomide (Len) and pomalidomide (Pom) and the PI bortezomib (Btz) and induced ADCP against MM cells that were sensitive and resistant to dexamethasone (Dex), IMiDs or daratumumab. In addition, VIS832 showed significant anti-cancer activity in a xenograft mouse model of MM, alone or in combination with Len or Btz, two drugs that synergize its therapeutic activity (161). In a previous work, Yu and colleagues investigated the use of the chimeric anti-CD138 mAb 1610 in *in vitro* studies and showed that it is able to induce ADCC cytotoxicity in CD138+ MM cells through NK cell-mediated activation (162).

#### 4.2.2 4B3

4B3 is a mouse mAb anti-CD138 that recognize a similar epitope with B-B4 and is capable to inhibit two type of MM cells (XG1 and XG-2) proliferation *in vitro* in a dose-dependent manner (163).

### 4.3 Antibody-drug conjugates

Antibody-drug conjugates (ADCs) are synthetic molecules that represent an innovative class of compounds with potent anti-cancer activity. Each ADC consists of a selective mAb, either full size or fragment, and a potent cytotoxic agent attached to the mAb through a linker, a short organic spacer. Compared to conventional chemotherapy, ADCs show remarkable improvements in terms of targeted killing of malignant cells and limited side effects outside the tumor, two aspects that strongly depend on their precise mechanism of action (178). Among the clinically recognized ADCs, belantamab mafotodine (Blenrep^®^) was recently approved by the FDA and EMA for the treatment of RRMM and targets the BCMA, a member of the tumor necrosis factor receptor family that is upregulated in this type of cancer (178). However, in 2022 Blenrep was withdrawn from market based on the outcome of the DREAMM-3 phase III confirmatory trial, which did not meet the requirements of the FDA Accelerated Approval regulations (www.gsk.com). Despite few ADCs targeting BCMA are currently in various stages of development, other candidate markers are also being investigated, including CD138.

#### 4.3.1 Indatuximab ravtansine

Indatuximab ravtansine or BT062 (Biotest AG) is an ADC consisting of a chimeric IgG4 mAb (clone B-B4) against CD138 conjugated via a disulfide-based linker to the maytansinoid drug DM4 (or Ravtansine), a microtubule polymerization inhibitor. The cytotoxicity is achieved by the dose-dependent activation of apoptosis. BT062 showed significant anti-cancer activity in preclinical testing, as it inhibited tumor growth and prolonged host survival when administered as monotherapy in xenograft mouse models (179) and in combination with Len and Len/Dex *in vitro* and *in vivo* mouse xenograft models of MM (153). Building on this promising foundation, a phase I clinical trial investigated the effect and best dose of BT062 when administered as monotherapy in RRMM patients (NCT00723359). This ADC was administered at a dose of 10 mg/m^2^ to 200 mg/m^2^ once every three weeks to 31 patients, all of whom received both a PI (Btz) and an IMiD (Len or Thal). The maximum tolerated dose (MTD) was set at 160 mg/m^2^ as few patients developed dose-limiting toxicities (DLT), including mucositis and skin and ocular toxicities, at this or a higher dose (200 mg/m^2^). Although the data from this study showed an acceptable safety profile, the low objective response rate (ORR) (3.2%) prompted the investigation of an alternative dosing regimen in a Phase I/IIa study (151). The clinical trial NCT01001442 enrolled 34 RRMM patients who had already received a PI (Btz) and an IMiD (Len or Thal) with the aim of evaluating the BT062 multi-dosage schedules. The data collected showed that the multi-dose regimen, administered on days 1, 8 and 15 of a four-week cycle, fixed the MTD at 140 mg/m^2^ and resulted in median PFS and OS of 3 and 26.7 months, respectively, with most adverse effects being grade 1 or 2, supporting further studies on the use of BT062 in combinatorial regimens (151). Following these encouraging results, a multicenter Phase I/IIa study was conducted in patients with RRMM to evaluate the safety, activity, and pharmacokinetics of BT602 and low-dose Dex in combination with the IMiDs drugs Len or Pom (NCT01638936). BT062 was administered intravenously on days 1, 8 and 15 of each 28-day cycle at increasing doses from 80 mg/m^2^ to 100 mg/m^2^ to 120 mg/m^2^. Data collected from patients receiving these drugs showed very similar median follow-up times (24.2 months for Len versus 24.1 months for Pol). In addition, the ORR for BT062 plus Len was observed in 71.7% of patients, while the ORR in the BT062 plus Pom group was 70.6% and the clinical benefit was 85% of patients treated with BT062 plus Len and 88% of patients treated with Pom, demonstrating the promising clinical activity of these combination treatments. In general, hematotoxicity (neutropenia in 22%, anemia in 16% and thrombocytopenia in 11% of patients) represented the main grade 3-4 adverse events in both groups, and treatment-related adverse events led to treatment discontinuation in almost half of the patients, while only a few of them resulted in non-BT062-related deaths. This study determined the MTD of BT062 plus Len at 100 mg/m^2^ and established the recommended Phase 2 dose for BT062 in combination with Pol (152).

#### 4.3.2 B-B4-DM1

B-B4-DM1 is an ADC composed of a IgG1 mAb anti-CD138 carrying the potent anti-microtubule polymerization agent DM1. B-B4-DM1 selectively reduced growth and survival of different CD138+ MM cell lines and in *in vivo* mouse models mediated promotion of MM regression, improvement in OS and reduction in the level of circulating human M protein at well tolerated doses (164).

### 4.4 Radioimmunoconjugates

Radioimmunoconjugates (or radiolabeled mAbs) consist of a mAb linked to a radionuclide. In contrast to ADCs, radioimmunoconjugates do not require internalization in the target cells to exert their anticancer effect, as the emitting radionuclide can also cause DNA strand breaks in the vicinity of the target. Moreover, depending on the length of the pathway traveled by the radionuclide, these compounds can also cause bystander killing (180).

#### 4.4.1 B-B4-I^131^

B-B4-I^131^ is a radiolabeled IgG1 mAb that binds to I^131^ and targets the extracellular domain of CD138. In a preliminary clinical study based on 4 RRMM patients who had received at least three prior lines of treatment, one patient experienced a partial response after administration of B-B4-I^131^, although the other three patients did not achieve a response and one of four patients suffered severe side effects after treatment (154). A Phase I clinical trial evaluating DLT to determine MTD in MM has been completed (NCT01296204). Other formulations labeling bismuth^213^ and lutetium^177^ have been studied in preclinical MM models. Chérel and colleagues showed promising therapeutic efficacy of 213Bi-labeled anti-mouse CD138 for the treatment of residual disease in MM, with only moderate and transient toxicity (166), though a study comparing B-B4-I^131^ and B-B4-Bi^213^ demonstrated that the latter showed significantly higher efficacy in terms of cell viability, blockade of G2/M phase and clonogenic survival in MM cell lines (165). In addition, Fichou and colleagues investigated the therapeutic efficacy of 9E7.4, an anti-mouse CD138 derivative radiolabeled with either bismuth-213 or lutetium-177, in an MM mouse model, showing little benefit for mouse survival (167).

### 4.5 Immunotoxins

Immunotoxins are immunoconjugates consisting of two functional subunits: a targeting system linked by a spacer to an effective complete or modified protein that serves as a payload. Interestingly, in addition to mAbs or small fragments, this component can also be a GF, a cytokine, or a chemokine (181). Toxins are highly potent proteins whose function is to disrupt vital cellular processes and cause cells death. They are mainly derived from bacteria, such as anthrax toxin, Shiga-like toxin, P. aeruginosa exotoxin A and diphtheria toxin, and from plants, such as pokeweed antiviral protein, saporin, ricin and gelonin, and must be engineered to remove the cell-binding domain and replace it with a targeting moiety (182, 183). As far as the use of immunotoxins in therapy is concerned, the main disadvantage lies in the nature of the toxins themselves, i.e. their immunogenicity triggers the production of antibodies against them (181, 184, 185). However, in order to increase their safety, the toxins can be modified to remove the epitope recognized by the immune cells and responsible of the unneeded immune reaction (184). Currently, 3 immunotoxins have been approved by the FDA for hematological malignancies (186). In MM, immunotoxins have been constructed from B-B2 and B-B4 mAbs by coupling them to the plant ribosome inactivating protein saporin, resulting in a significant reduction in the number of PCs (168).

### 4.6 Immunocytokines

Immunocytokines are fusion proteins made of Abs and cytokines specifically designed to interact with dysregulated antigens that are expressed to a greater extent on tumor cells or in the TME. This formulation is designed to target a cytokine receptor on T or NK cells to trigger a targeted immune response against cancer cells (181, 187). To date, only interferon-α (IFNα) and IL-2 have been approved by the FDA for cancer treatment (188). 3 immunocytokines combining the specificity of IgG1 anti-CD138 mAb with the cytokines IFNα14, IFNα2 or IFNα2YNS, a mutated moiety, were investigated regarding their use as a therapeutic approach in MM. *In vitro* analyses performed on MM cell lines showed an intriguing cytotoxic effect of all formulations, alone or in combination with PI Btz. Moreover, *in vivo* experiments demonstrated their anticancer activity by reducing MM tumor growth and prolonging survival, highlighting their potential as new and innovative candidates for MM treatment (169, 170, 189).

### 4.7 Recombinant proteins and bispecific antibodies

Recombinant proteins are defined as large and complex molecules whose therapeutic activity is highly dependent on their structure (190, 191). Bispecific antibodies (BsAbs) are emerging immunotherapeutics designed to ideally achieve a stronger benefit than the solely mAbs. BsAbs interact with both an antigen on the malignant cell population and a second target on immune effector cells, such as T and NK cells. In this manner, this T cell receptor (TCR)-independent interaction results in activation of T/NK cells and therefore causes direct lysis of tumor cells. To date, all BsAbs in Phase I/II clinical trials use CD3, a T cell co-receptor, to activate T cells, but none have been approved for the treatment of MM yet (32, 192).

#### 4.7.1 ULBP2-BB4

ULBP2-BB4 is a recombinant bispecific protein designed to induce NK cells toxicity on CD138+ cell lines, including MM cells. This recombinant compound consists of the anti-CD138 mAb B-B4 fused to UL-binding protein 2 (ULBP2), a ligand capable of binding the NKG2D receptor, which is mainly expressed on NK cells. While BB4 targets the CD138+ MM cells, ULBP2 stimulates the NKG2D receptor on NK cells and triggers the release of soluble factors involved in NK recruitment and activation of cytotoxicity. Interestingly, ULBP2-BB4 showed potent anti-tumor activity *in vitro* and inhibited tumor growth in a mouse model of MM (171).

#### 4.7.2 STL001 (or BiTE-hIgFc)

STL001 is a bispecific anti-CD138 x anti-CD3 Ab consisting of two single chain variable fragment (scFV) arms with the IgG1-Fc region sequence. This bispecific T cell engager (BiTE) can target immune T and NK cells as well as MM cell co-cultures and showed stronger anti-tumor activity *in vitro* than the combination of single anti-CD138 and anti-CD3 mAbs. Notably, STL001 achieved lysis of 90.1% of MM cells, remarkable T cell activation efficiency and phenomenal interaction with NK cells *in vitro*. In addition, administration of STL001 in a human MM xenograft mouse model resulted in a significant 75% reduction in tumor volume compared to the negative control (172).

#### 4.7.3 h-STL002 and m-STL002

In addition to STL001, Chen and colleagues have developed two recombinant BsAbs targeting CD138 and CD3, the co-receptor involved in T cell activation, consisting of two scFv arms and an IgG1-Fc region. Preclinical data from experiments with MM cell co-cultures showed strong cytotoxicity in this *in vitro* model via a remarkable T cell-mediated immunological response (173).

### 4.8 CAR-T cells

Chimeric antigen receptor (CAR) T cells are genetically engineered T cells derived from MM patients that have been modified to express the chimeric protein against the selected antigen. In this sense, CAR-T cells express an extracellular synthetic mAb-derived scFv fused with co-stimulatory intracellular domains to trigger the activation and redirection of T cells upon tumor binding to enable cancer cell recognition and suppression (193).

#### 4.8.1 CART-138

CART-138 are modified CAR-T cells consisting of the scFv against CD138 fused to the T cell activation domain 4-1BB. In the phase I/II of NCT01886976 clinical trial, autologous CART-138 was tested as a therapeutic option in 5 patients with chemotherapy-refractory MM. The data collected in this study showed that the 4 infused patients reentered into a stable disease state for more than three months, and 1 patient with advanced plasma cell leukemia experienced a significant reduction of MM cells in his peripheral blood. Along with the clinical outcome, the treatment regimen was generally safe and well tolerated and caused no serious adverse effects (155).

#### 4.8.2 CD138.CAR

The investigation of the feasibility of CD138 in CAR-T therapy led to the development of an alternative CAR-T-based strategy. Sun and colleagues developed CD138-specific CAR cells (CD138.CAR) expressing the scFv of the chimeric mAb BT062 together with various signaling (CD28, 4-1BB) and endodomains (CD28, CD8a). In their study, they showed that CD138.CARs can be expressed by T cells from healthy donors and that they target CD138+ MM cell lines while sparing normal epithelial and endothelial cells. In addition, the authors have shown that these CAR-T cells can be derived from MM patients and are directed against autologous CD138+ MM cells and putative MM cancer stem cells, ultimately exhibiting significant anti-cancer activity in a xenograft mouse model of MM. Similar to the previous study, infusion of the engineered T cells was generally safe and well tolerated, suggesting that therapeutic strategies based on CAR-T cells may have the potential to achieve remarkable results in MM patients (174).

#### 4.8.3 CAR-NK

In addition to reprogramming T cells, Jiang and colleagues reprogrammed NK cells with a CAR to generate 4B3 anti-CD138 scFv fused to the CD3ζ chain as a signaling moiety. These CAR-NK cells showed increased anti-MM cytotoxicity against CD138+ MM cell lines and achieved promising results in the xenograft NOD-SCID mouse model (175).

### 4.9 Peptide and vaccines

Peptides are based on *in vitro* synthesized peptides with 8–30 amino acids, which are known to be highly immunogenic and trigger the desired immune response. Vaccines, which can be defined as a cocktail of peptides, can keep the body’s immune effector cells in a constant active state to keep the tumor under attack (194).

#### 4.9.1 GLVGLIFAV peptide and PVX-410 vaccine

GLVGLIFAV (also known as L-valine-glycyl-l-leucyl-l-valylglycyl-l-leucyl-l-isoleucyl-l-phenylalanyl-l-alanyl- or CD138_260–268_) is a short and immunogenic human leukocyte antigen A2 (HLA-A2)-specific CD138 epitope nanomer, which elicits a restricted cytotoxic T lymphocyte (CTL) response to MM cells positive to CD138 and HLA-A2 expression (195). The efficacy of the GLVGLIFAV peptide was demonstrated by its ability to induce CD138-CTL anticancer activity against primary CD138+ cells isolated from HLA-A2+ MM patients and by the high level of intracellular IFNγ and cell proliferation in response to MM cell lines (195). The ability to elicit more specific CTLs response was evaluated with PVX-410, a cocktail of HLA-A2-specific peptides and including CD138_260–268_, using T cells from SMM patients. Interestingly, data revealed an effective anti-MM response in an HLA-A2-restricted and peptide-specific manner (196). The combination of PVX-410 vaccine with or without Len in SMM patients was assessed in a phase I/IIa non-randomized clinical trial (NCT01718899) (156). The data collected in this study showed that the multipeptides vaccine PVX-410 elicited a highly effective immune response against MM cells by expanding CD3+ CD8+ CTL components against CD138 and other epitopes. Moreover, PVX-410 was safe and well tolerated when administered as monotherapy and even more effective when combined with lenalidomide (156). In line with this study, a phase I clinical trial aims to test the effect of PVX-410 vaccine with citarinostat, a histone deacetylase inhibitor, with/without Len in SMM patients (NCT02886065).

### 4.10 Synstatins

Synstatins (SSTNs) are short inhibitory peptides derived from specific strings of amino acid in the CD138 extracellular domain. Synthetic SSTNs, by mimicking few selected sites on CD138 ectodomain, shall compete with agonists receptor binding to their docking motifs, thus blocking their pro-tumor signaling cascades (176).

#### 4.10.1 SSTN_IGF-1R_

CD138 has been shown to be an excellent antigen to be targeted by SSTNs as it plays a role in tumorigenesis together with insulin-like growth factor receptor (IGF-1R) type I and tumor-induced integrins αvβ3/αvβ5 (176, 177). In response to PCs malignant transformation, overexpression of integrins leads to the clustering of pre-assembled inactive CD138-αvβ3/αvβ5-IGF-1R ternary complex and, upon engagement of CD138 to ECM-ligands, the IGF-1/2-independent triggering of IGF-1R. By inactivating ASK-1 and the associated suppression of JNK pro-apoptotic activity, MM cells support their survival and, via Talin-directed inside-out integrin signaling, also angiogenesis and invasiveness. SSTN_IGF-1R_ (or SSTN_92-119_) mimics the docking site of αvβ3 and αvβ5 integrins and IGF-1R to prevent their interaction with CD138, thereby competitively disrupting the ternary complex and smothering pro-tumor pathways (121, 176, 177).

#### 4.10.2 SSTN_VLA4_ and SSTN_VEGFR2_

SSTN_VLA4_ and SSTN_VEGFR2_ are two peptides whose sequences mimic the docking motif for VLA4 and VEGFR2, respectively, at a juxtamembrane region from amino acid 210 to 236 in the extracellular domain of CD138. Specifically, SSTN_VLA4_ is shorter than the other SSTN and carries an essential DFTF domain to its N-terminus to enable binding of VLA4, while SSTN_VEGFR2_ mimics the CD138 docking motif of VEGFR2 and carries the important PVD domain at its C-terminus (116, 176, 177). In malignant PCs, the invasive phenotype promoted by CD138-mediated activation of VLA4 can be blocked by SSTN_VLA4_, preventing VLA4 docking on transmembrane CD138. On the contrary, the need to block VEGFR2 comes from its role in cell invasion and polarization. After truncation of HS polymers by HPSE and the shedding by MMP-9, soluble CD138 couples to an inactive complex formed by various proteins, including VEGFR2, leading to its ligand-independent activation. This event triggers an intracellular cascade that leads to enhanced VLA4-madiated invasion of immune and MM cells, but also inhibits LFA-1-mediated migration of NK and CD8+ T tumor suppressor cells. In this context, SSTN_VEGFR2_ serves to prevent VEGF2-independent activation of this pathway by sequestering secreted CD138, thereby targeting key processes of tumorigenesis (116, 176, 177).

### 4.11 Heparanase inhibitor

HPSE is the main enzyme responsible for extracellular HS degradation and its expression is significantly increased in aggressive cancers, facilitating tumor progression through metastatic dissemination (122, 197). Given the central role of HPSE in CD138 metabolism and in the progression of MM, it represents a promising candidate for the development of new antitumor agents.

#### 4.11.1 Roneparstat

Roneparstat (or SST0001) is a polymer with a heparin-like structure that is able to inhibit the growth and angiogenesis of MM by disrupting the HPSE-CD138 axis (198). As Roneparstat achieved significant anti-MM activity in preclinical mouse models in monotherapy or in combination with Dex, Btz or melphalan, its safety dose and tolerability were assessed in an open-label, multicenter, phase I clinical trial enrolling RRMM patients (NCT01764880), with little efficacy (157).

## 5 Conclusions

In recent years, several advances have been made in improving therapeutic modalities for MM, including the discovery of new drugs and novel supportive care. However, most patients still die from their cancer since MM remains highly heterogeneous and largely incurable. The effects of immunological agents, such as mAbs, BiTEs and CAR-Ts, which are now widely used in clinical practice, are dependent on the expression of specific antigens on MM cells. However, it is difficult to monitor the expression of these antigens accurately and consistently in practical treatment. As therapy progresses, the number of cell clones that do not express a specific antigen gradually increases, and the development of immune escape mechanisms eventually leads to drug resistance. Together with other HPSGs, CD138 acts as a critical regulator in the communication between malignant PCs and the BMME, fueling MM tumorigenesis and progression. In this sense, by mediating the activity of specific GFs from the BM niche, CD138 triggers key pathways required by MM cells for their homing, growth, and survival. The surface proteome of MM cells regulates PCs biology and delineates therapy targets. As recently reported, CD138 has been detected as one of the most important antigens characterizing the surfaceome of MM cells, along with the canonical and better-studied markers BCMA, CD38 and SLAMF7 among others (199). Accordingly, most preclinical and clinical MM studies to date have focused on the key immunotherapy antigens BCMA, CD38 and SLAMF7, with the latter two being the target of three already FDA-approved mAbs. BCMA, CD38 and SLAMF7 represent three key antigens in the biology of MM; the first plays a key role in B cell maturation and differentiation into PCs and is upregulated in disease progression from MGUS to SMM and active MM (15); the second is uniformly expressed on PCs and is involved in modulating immune cell activation and migration (9), while the latter is involved in the development of immune system and promotes PCs proliferation and growth (13). Given its central role in MM biology, restricted expression on normal PCs but strong upregulation on malignant cells, BCMA has become the most common target of various therapeutic strategies, each associated with logistical challenges and unique toxicities. Among the most interesting BCMA-based approaches investigated are BiTEs, which showed high efficacy and moderate toxicity but short half-life, CAR-Ts, which achieved high response rates in heavily pretreated MM patients but induced cytokine release syndrome (CRS) and increased susceptibility to infection, and ADCs, which exhibited corneal toxicities and need further investigation (181, 192, 200–202). While BCMA expression increases significantly with the progression of MM, but the intensity of expression varies greatly from patient to patient (203) potentially affecting the therapeutic benefits, CD38 levels are robust and constant during MM pathogenesis. In addition to daratumumab and isatuximab, two FDA-approved mAbs for the treatment of newly diagnosed (ND)/RRMM patients, CD38 is largely targeted by the development of BsAbs and immunoconjugates, some of which are in preclinical and clinical stages, as well as mono/dual-specific CAR-Ts, the latter with high efficacy and safety (204). However, the simultaneous expression of CD38 on healthy PCs and other hematopoietic (T lymphocytes) and non-hematopoietic cells, such as epithelial, endothelial and vascular smooth muscle cells, as well as hepatocytes, goblet and columnar cells of gastrointestinal tract (205) may pose a serious obstacle to its use as a targeted MM antigen, as it may likely cause severe off tumor side effects in the already susceptible MM patients. In this context, the ADC BT062 led to skin and mucosal toxicities, the radioimmunoconjugate B-B4-I^131^ caused severe side effects in one patient after treatment, and CRS and neurotoxicity mediated by proinflammatory cytokines upon CAR-T cell activation represent another issue to be addressed, although no significant toxicities were observed in a small pilot study with CD138 CAR-T cells (206, 207). Nevertheless, the likelihood of on-target/off-tumor side effects should be expected as key MM antigens are also expressed on normal cells and therapies based on these antigens could lead to common manifestations and severe toxicities (207). Apart from the comparable importance between these antigens and CD138 in this type of cancer, this marker has gained considerable attention in the therapeutic panorama of MM as it shares important functional features with the promising candidates mentioned above, including significant presence and easy accessibility on the malignant cell membrane. Most importantly, CD138 is often abundant even in RR stages of MM, making it a consistent target for therapy. In this context, while there are some fractions of MM cells that do not express CD138 or express it at low levels, which may impact the efficacy of antigen-based molecules, the fact remains that such therapies may increase the chances of thorough eradication of all malignant clones in more aggressive stages of MM disease, as these are characterized by higher levels of CD138, as evidenced by higher serum levels in these patients. Nevertheless, since the CD38 marker may fade or weaken compared to the CD138 marker in PCs after chemotherapy (208) and BCMA- and/or CD38-negative relapses have also been observed (209–211), the need for complementary treatment strategies must be carefully considered and strongly recommended, highlighting CD138 as a compelling candidate for further development of anti-MM approaches. Significant efforts have been made to identify therapeutic treatments against CD138. In this sense, many innovative anti-CD138 immunotherapeutics developed by engineering and/or arming mAbs with toxic payloads have provided interesting results in terms of PC targeting, therapeutic efficacy, and safety in different models, with ADC Indatuximab ravtansine probably being the most interesting candidate for future clinical application. Other studies have provided robust proof of concept for MM cell killing by simultaneously targeting CD138 and specific receptors on T/NK cells to trigger their activation, with promising results in cell lines, in mouse models and in MM patients as well. In addition, new avenues have recently been explored focusing on inhibitory peptides that either induce the cytotoxic anti-cancer activity of CTLs or mimic key interacting domains on CD138, as well as polymers that offer the possibility of disrupting molecular interactions useful for MM progression leading to anti-cancer activity. However, for most approaches, the still limited amount of data suggests further efforts to better characterize these compounds and their relationship with the cellular environment. Although most CD138-targeting strategies have shown promising results in *in vitro* and/or *ex vivo* cells and in mouse models, there are still some caveats and limitations that need to be carefully considered, especially in terms of mechanism of action and safety. In addition, since few immune- and peptide-based approaches have been tested in combination with IMiDs and PIs in MM patients, extensive efforts are underway to identify additive (or even synergistic) benefits when combined CD138-based treatments with these drugs and non-overlapping toxicity profiles that could enable tolerable and novel combination therapies.

## Author contributions

FR: Investigation, Writing – original draft, Writing – review & editing. CT: Writing – review & editing, Writing – original draft. MDB: Funding acquisition, Supervision, Writing – review & editing. GT: Funding acquisition, Supervision, Writing – review & editing.
